# Analysis of gene expression during the transition to climacteric phase in carnation flowers (*Dianthus caryophyllus* L.)

**DOI:** 10.1093/jxb/ert281

**Published:** 2013-09-28

**Authors:** Byung-Chun In, Brad M. Binder, Tanya G. Falbel, Sara E. Patterson

**Affiliations:** ^1^Department of Horticulture, University of Wisconsin-Madison, Madison, WI 53706, USA; ^2^Department of Biochemistry, Cellular, and Molecular Biology, University of Tennessee, Knoxville, TN 37996, USA

**Keywords:** Carnation, ethylene biosynthesis, ethylene receptors, 1-MCP, petal inrolling, senescence.

## Abstract

It has been generally thought that in ethylene-sensitive plants such as carnations, senescence proceeds irreversibly once the tissues have entered the climacteric phase. While pre-climacteric petal tissues have a lower sensitivity to ethylene, these tissues are converted to the climacteric phase at a critical point during flower development. In this study, it is demonstrated that the senescence process initiated by exogenous ethylene is reversible in carnation petals. Petals treated with ethylene for 12h showed sustained inrolling and senescence, while petals treated with ethylene for 10h showed inrolling followed by recovery from inrolling. Reverse transcription–PCR analysis revealed differential expression of genes involved in ethylene biosynthesis and ethylene signalling between 10h and 12h ethylene treatment. Ethylene treatment at or beyond 12h (threshold time) decreased the mRNA levels of the receptor genes (*DcETR1*, *DcERS1*, and *DcERS2*) and *DcCTR* genes, and increased the ethylene biosynthesis genes *DcACS1* and *DcACO1*. In contrast, ethylene treatment under the threshold time caused a transient decrease in the receptor genes and *DcCTR* genes, and a transient increase in *DcACS1* and *DcACO1*. Sustained *DcACS1* accumulation is correlated with decreases in *DcCTR* genes and increase in *DcEIL3* and indicates that tissues have entered the climacteric phase and that senescence proceeds irreversibly. Inhibition of ACS (1-aminocyclopropane-1-carboxylic acid synthase) prior to 12h ethylene exposure was not able to prevent reduction in transcripts of *DcCTR* genes, yet suppressed transcript of *DcACS1* and *DcACO1*. This leads to the recovery from inrolling of the petals, indicating that *DcACS1* may act as a signalling molecule in senescence of flowers.

## Introduction

Petal senescence is the final event in floral development and is mediated by a sequence of highly controlled physiological and biochemical changes that are regulated by ethylene in many flower species (Halevy and Mayak, 1981; van Doorn and van Meeteren, 2003). In ethylene-sensitive flowers such as carnations, ethylene perception is an indispensable requirement to initiate and sustain the ethylene-mediated senescence programme (Thomas *et al.*, 1985; Borochov and Woodson, 1989). When ethylene is perceived by the receptors, the ethylene signal is sent through a sequence of biochemical events that regulate the expression of ethylene-responsive genes leading to ethylene biosynthesis and ultimately senescence of flowers. The ability to perceive or respond to ethylene is most probably mediated by changes in ethylene signalling during flower development (Halevy and Mayak, 1981; Woodson and Lawton, 1988; Verlinden *et al.*, 2002).

Carnation flowers show characteristic floral senescence patterns in response to ethylene. An inward rolling of the petals (petal inrolling) occurs shortly after exposure to ethylene, prior to the initiation of petal wilting; this is regarded as an initial morphological indicator of senescence in carnations (Halevy and Mayak, 1981; Kim *et al.*, 1998; Reid and Çelikel, 2008). Carnation petal senescence appears to be under the control of autocatalytic ethylene biosynthesis as it coincides with the climacteric rise in ethylene synthesis (Halevy and Mayak, 1981; Wang and Woodson, 1989).

Ethylene biosynthesis in plants is under stringent metabolic regulation during development and senescence. The conversion of *S*-adenosylmethionine (SAM) to 1-aminocyclopropane-1-carboxylic acid (ACC) by ACC synthase (ACS) members is the first rate-limiting step in the ethylene biosynthesis pathway. Subsequent to this conversion, ACC is converted to ethylene by ACC oxidase (ACO) members (Yang and Hoffman, 1984; Kende, 1993; Schaller and Kieber, 2002). It has been proposed that there are two different systems of ethylene synthesis: system 1 is responsible for the basal low level of ethylene production (pre-climacteric phase), detectable in all plant organs, and functions during plant growth and development; and system 2 mediates autocatalytic ethylene production (climacteric phase) and functions during flower senescence and fruit ripening (McMurchie *et al.*, 1972; Yang and Hoffman, 1984; Kende, 1993). Transition of tissues to the climacteric phase during floral senescence in carnation has been shown to be accompanied by changes in transcripts of ethylene biosynthesis genes (Woodson *et al.*, 1992; ten Have and Woltering, 1997; Verlinden *et al.*, 2002; Jones, 2003).

Autocatalytic ethylene synthesis during petal senescence occurs as a result of changes in the ethylene sensitivity of the tissues, and the exposure of pre-climacteric petals to exogenous ethylene can lead to transition to autocatalysis of ethylene synthesis (Mayak and Kofranek, 1976; Halevy and Mayak, 1981; Wang and Woodson, 1989). Previous work has concluded that once petal tissues have entered into this autocatalytic synthesis of ethylene, the senescence process is generally irreversible (Halevy and Mayak, 1981; Yang and Hoffman, 1984). However, in a previous study of carnations (*Dianthus caryophyllus* L. ‘Glacier’), petals exhibited a reversible senescence process.

In this work, the molecular events and ethylene levels that are associated with the climacteric response were studied. Pre-climacteric petal tissues have a lower sensitivity to ethylene and, at a critical point, this shifts the tissues to start autocatalytic ethylene synthesis. The relationship between the change in ethylene sensitivity and senescence of petals was characterized by monitoring petal inrolling and recovery patterns in response to different doses of ethylene. A molecular approach was also taken to explore the genes involved in the transition from the pre-climacteric to climacteric phase. As part of this approach, the mRNA levels of genes involved in ethylene biosynthesis and signalling were monitored, to test the hypothesis that specific genes in ethylene signalling may suppress the accumulation of the ethylene biosynthesis genes, and inactivation of the genes by constitutive perception of ethylene may induce accumulation of the biosynthesis genes.

## Materials and methods

### Plant materials

Rooted cuttings of carnation plants (*D. caryophyllus* L. cv. Glacier) were planted in 15cm plastic pots in growing medium (Metro-Mix Special Blend, SUNGRO Horticulture Distribution Inc., Bellevue, WA, USA). Plants were grown in a greenhouse at 22/16 °C day/night temperature and drip-irrigated every other day with half-strength Hoagland nutrient solutions. Supplementary lighting [220 µmol m^–2^ s^–l^ photosynthetic photon flux density (PPFD) at plant level] was provided by high-pressure sodium lamps (Philips 600W Master GreenPower) to ensure a photoperiod of 16h. These lamps were turned on automatically when the intensity of natural lighting was <350 µmol m^–2^ s^–l^. Plants were pinched 4 weeks after planting and transplanted into 30cm plastic pots. After harvest, carnation flowers were immediately placed in tap water for all experiments.

### Kinetic analysis for inrolling and angle of petals

The outer whorl of petals was detached from fully open flowers by carefully pulling them out from the receptacles and the individual petals were immediately placed in 1.5ml micro tubes containing distilled water. To measure petal inrolling due to ethylene, petals were enclosed in a transparent plastic chamber (25 litres) with air circulation by a small fan at 21 °C under light conditions (10 µmol m^–2^ s^–1^). Ethylene was injected into the chamber to give a final concentration of 10 µl l^–1^ and withdrawn from the treatment chamber after 10h of incubation. During incubation of the petals, the front and side of the petals were photographed with a digital camera at 1h intervals for 6 d. The diameter and angle of petals were analysed from the images using the software ImageJ (http://rsb.info.nih.gov/ij). To compile graphs showing the changes in petal angle, the angle was defined as 0 ° when the petal stands vertically, –90 ° when the petal is perpendicular to 0 ° in an anticlockwise direction, and 90 ° when the petal is perpendicular to 0 ° in a clockwise direction.

### Ethylene treatment and measurement of petal diameter

To determine the effect of ethylene treatment time on the inrolling, petals were enclosed in a plastic chamber (117 litres) with air circulation by a small fan at 21 °C under dark conditions. Ethylene was injected into the chambers to give a final concentration of 10 µl l^–1^ and the petals were incubated under ethylene atmosphere for various durations (9, 10, 11, and 12h). For treatment with various doses of ethylene, petals were incubated in the treatment chambers with 1, 10, or 100 µl l^–1^ ethylene for 10h.

**Fig. 1.**
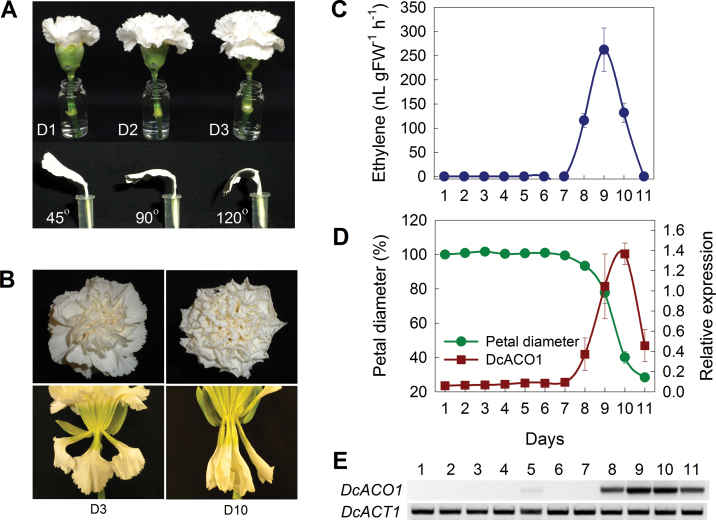
Petal inrolling during flower senescence in carnation cv. Glacier. (A) An open flower with fully expanded and turgid petals and a senescent flower with completely inrolled petals. (B) Petals detached from carnation flowers at three distinct stages in flower development: day 1 (D1), flowers beginning to open; day 2 (D2), flowers opened; and day 3 (D3), flowers fully opened with appearance of the stigma. The stages of the petals were defined as 45, 90, and 120 ° based on the degree of petal angles with respect to the axis of the pedicle. (C) Ethylene production by whole flowers during natural senescence. (D) Petal inrolling and relative expression level of *DcACO1* during natural senescence of detached petals. (E) Expression profiles of *DcACO1* and *DcACT*1.

For ethylene treatment on the petals at different stages, the outer petals were detached from carnation flowers at three distinct stages of flower development: day 1 (D1), flowers beginning to open; day 2 (D2), flowers fully opened; and day 3 (D3), flowers fully opened with appearance of the stigma (Fig. 1A). The stages of the petals were defined as 45 °, 90 °, and 120 ° based on the degree of the angles of the outer petals with respect to the axis of the pedicle (Fig. 1A). The petals in three different stages were incubated in the treatment chamber with 10 µl l^–1^ ethylene for 10h. Untreated petals were incubated in the same chambers with normal air and used as control.

Ethylene treatment was terminated by transferring the petals from the treatment chambers to normal atmospheric air. After the treatments, the petals were held in the laboratory environment at 21±2 °C, 30±10% relative humidity, and ambient laboratory fluorescent lighting at ~10 µmol m^–2^ s^–1^. Petal diameter was measured using digital calipers before ethylene treatment (–12h) and at 0 (20min in air), 4, 8, 12, 16, 24, and 48h following ethylene removal.

### Treatment with 1-methylcyclopropene (1-MCP)

The ethylene binding antagonist, 1-MCP was applied via SmartFresh (AgroFresh Inc., Philadelphia, PA, USA) tablets in the treatment chamber at 21 °C under dark conditions. To generate 100 nl l^–1^ 1-MCP gas, a grey research tablet (0.026mg 1-MCP) and an activator tablet was added to a beaker containing 18ml of activator solution in the chamber. The tubes with petals were transferred to the chamber along with a small fan to provide air circulation during the treatment and then the chamber was tightly sealed for 8h. After the treatments, the petals were held in the laboratory environment. For 1-MCP treatment following 10 µl l^–1^ ethylene exposure, carnation petals (90 °) were treated with 100 nl l^–1^ 1-MCP for 8h and kept under normal atmospheric air.

### Inhibition of ethylene biosynthesis

Carnation petals (90 ° stage) were treated either simultaneously with aminoethoxy-vinylglycine (AVG; Sigma-Aldrich Co., USA) and ethylene or with AVG prior to ethylene. For the simultaneous treatment, the petals were placed in the tubes containing 100 µM AVG solution and simultaneously incubated in the treatment chamber with 10 µl l^–1^ ethylene for 12h. AVG treatments prior to ethylene began 8h before ethylene exposure. After ethylene treatments, the petals were subsequently treated with 100 nl l^–1^ 1-MCP or air for 8h. Once AVG treatment started, all petals were kept in the AVG solution throughout the experiment.

Treatments with AVG and ACBC (1-aminocyclobutane-1-carboxylate) (Tocris Bioscience, UK), an inhibitor of ACC oxidase, were carried out before ethylene exposure. The petals were held in tubes containing 100 µM AVG or a mixture of 100 µM AVG plus 100 µM ACBC for 12h and subsequently treated with 10 µl l^–1^ ethylene for 12, 14, or 20h.

### Ethylene measurements

Three carnation petals in each treatment were selected and individual petals were enclosed in 25ml glass vials for 1h at 21 °C. Gas samples (1ml) were collected with a gas-tight hypodermic syringe through a rubber septum and analysed for ethylene by gas chromatography (Model 8500, Perkin Elmer Corp., Norwalk, CT, USA) equipped with an aluminium column and flame ionization detector. The detector and oven temperature of the gas chromatograph were held 170 °C and 120 °C, respectively. N_2_ was used as a carrier gas at a flow rate of 20ml min^–1^. Each experiment was repeated at least three times.

### cDNA synthesis and semi-quantitative RT–PCR

Total RNA was isolated from various floral organs using the Trizol method according to the manufacturer’s procedure with slight modifications. Petals were collected before ethylene treatment (–12h) and at 0 (20min in air), 8, 24, and 48h following ethylene removal and immediately frozen in liquid N_2_ and stored at –80 °C until RNA isolation. Individual petals (~300 µg) were ground in the presence of liquid nitrogen to a fine powder using a pre-chilled mortar and pestle and homogenized in 1ml of Trizol reagent (Invitrogen, Carlsbad, CA, USA). These samples were separated into aqueous and hydrophobic phases with 200 µl of chloroform by centrifugation. The RNA was precipitated from the aqueous phase with 50 µl of 5M NaCl and 0.5ml of isopropanol, washed with 80% ethanol, and resuspended in RNase-free H_2_O. Total RNA was quantified with a NanoDrop DN-1000 spectrophotometer (NanoDrop Technologies Inc., Wilmington, DE, USA). RNA samples were treated with RNase-free DNase prior to RT–PCR, and first-strand cDNA was synthesized from 2 µg of total RNA with 1 µg of oligo(dT)_18_ primer, dNTPs, RNA inhibitor, buffer, and M-MLV reverse transcriptase in a final volume of 25 µl according to the manufacturer’s instructions (Promega, Madison, WI, USA). The reverse transcription was performed in a PTC-200 PCR machine (MJ Research Inc., MA, USA) with the following temperature parameters: 15min at 70 °C followed by 1h at 42 °C.

Gene-specific primers were designed for the ethylene biosynthesis genes (*DcACS1*, *DcACS2*, *DcACS3*, and *DcACO1*) and ethylene signalling genes (*DcETR1*, *DcERS1*, *DcERS2*, *DcCTR1*, *DcCTR2*, *DcEIL1*, *DcEIL2*, *DcEIL3*, and *DcEIL4*) and synthesized by Integrated DNA Technologies (Coralville, IA, USA). Carnation actin (*DcACT1*) was used as an internal control. Primer pairs used for semi-quantitative PCR analysis are listed in Table 1. For PCRs, 1 µl of cDNA was used as a template with 2 µl of PCR buffer, 0.2 µl of *Taq* polymerase, dNTPs, and forward and reverse primers in a final volume of 20 µl. PCR amplification was performed with the following temperature parameters: 95 °C for 3min and then cycled 35 or 40 times at 95 °C for 20 s, 60 °C for 30 s, and 72 °C for 40 s, with a final extension of 7min at 72 °C. The PCR products were then analysed using a 1% (w/v) agarose gel and stained with ethidium bromide. The gels were visualized under UV light and the images were taken using a gel documentation system (Gel Dec XR+, Bio-Rad, Hercules, CA, USA) and quantified with ImageJ 1.43u (National Institutes of Health, Bethesda, MD, USA). The relative level of the band was shown as the absolute integrated absorbancy normalized to the relative actin band. All experiments were performed with three independent biological replicates.

**Table 1.** Gene-specific primers used for RT–PCR amplification of cDNA fragments

Gene	Accession number	Forward primer	Reverse primer	Cycle
*DcSAMS1*	M81882.1	5′-GACCAGGGTCACATGTTCG-3′	5′-TCGTGGATCTTTCCTGTTCC-3′	35
*DcACS1*	M66619.1	5′-AAAATCGCTACGAACGATGG-3′	5′-TGCGGGATAATAAGGAGACG-3′	40
*DcACS2*	AF049138.1	5′-TTTACTAGGGGGCCTTAGCC-3′	5′-CTTAGTAGACGCCACGACAGG	40
*DcACS3*	AF049137.1	5′-TAGGGTTTCCGGGATTTAGG-3′	5′-ATCCTCCAATCGTCTGTTCG-3′	40
*DcACO1*	AB042320.1	5′-TCACAACTGGGGATTCTTCC-3′	5′-CATCTGTCTGCGCTATCACG-3′	40
*DcETR1*	AB035806.1	5′-CACACTCCGTCAGCAATATCC-3′	5′-TCCATAAGCTGTTGCTGTGC-3′	40
*DcERS1*	AF016250.1	5′-CGTCTGTGCTGTAGGTGACG-3′	5′-CAAATTGCGAGTCCAAGTCC-3′	40
*DcERS2*	AF034770.1	5′-AGAGTGACGGTCATGACAAGG-3′	5′-CCTAACCGCGCTTAACTCC-3′	35
*DcCTR1*	AF261147.1	5′-TTCAGGGACTGCTGAGTCG-3′	5′-ATGCCTACAGAGGCCAACC-3′	40
*DcCTR2*	AF261148.1	5′-ATTCGCTGTCTCGATTGTCG-3′	5′-GCTCTGCAGTTCTTTTAATTTGG-3′	35
*DcEIL1*	AF261654.1	5′-TGCAGGAACTTCAAGACACG-3′	5′-AGTCTGAGATCCCGATGACG-3′	40
*DcEIL2*	AY728191.1	5′-GCTGGCGATCATAAATCAGG-3′	5′-TCTTACGCTGCATGTTCTGC-3′	40
*DcEIL3*	AY728192.1	5′-AACTCAACCCGTGATTCTGC-3′	5′-GCACATCATCCATGAAATCG-3′	35
*DcEIL4*	AY728193.1	5′-CTCGGAGGAAGAAGATGTCG-3′	5′-TGATCACGGCTGTTAGAACG-3′	40
*DcACT1*	AY007315.1	5′- CGTCACCAACTGGGATGACA-3′	5′- GAGAGAACGGCCTGGATGGC-3′	35

### Phylogenetic analysis

Protein sequences for the carnation EIL family (*DcEIL1–DcEIL4*), and *Arabidopsis* EIN3 (*AtEIN3*) and EIL1 (*AtEIL1*) were obtained from the GenBank database (NCBI) and aligned by using ClustalX (version 1.83). Based on the protein sequence similarities, an unrooted Neighbor–Joining phylogenetic tree was constructed by using MEGA software (version 5.05).

## Results

### Petal inrolling petal angle and recovery patterns

In carnation ‘Glacier’ flowers, three distinct stages can be distinguished during flower opening (D1, D2, and D3) and the outer petals show 45 °, 90 °, and 120 ° angles, respectively (Fig. 1A). The fully opened flowers (D3) at 3 d after harvest show turgid and fully expanded petals, while the senescent flowers (D10) 10 d after harvest are completely wilted and display inrolled petals (Fig. 1B). Ethylene production in whole flowers rapidly increases at the later stages and peaks at day 9 (Fig. 1C). *DcACO1* expression in petals shows a similar pattern to the ethylene production in whole flowers. *DcACO1* transcript greatly increases on day 9 when the petal diameter starts to decrease, and then rapidly decreases coincident with a complete inrolling of petals (Fig. 1D, E). The onset of petal inrolling is accompanied by a significant increase in *DcACO1* and ethylene synthesis, indicating that petal inrolling is an excellent morphological indicator of senescence in carnation ‘Glacier’.

**Fig. 2.**
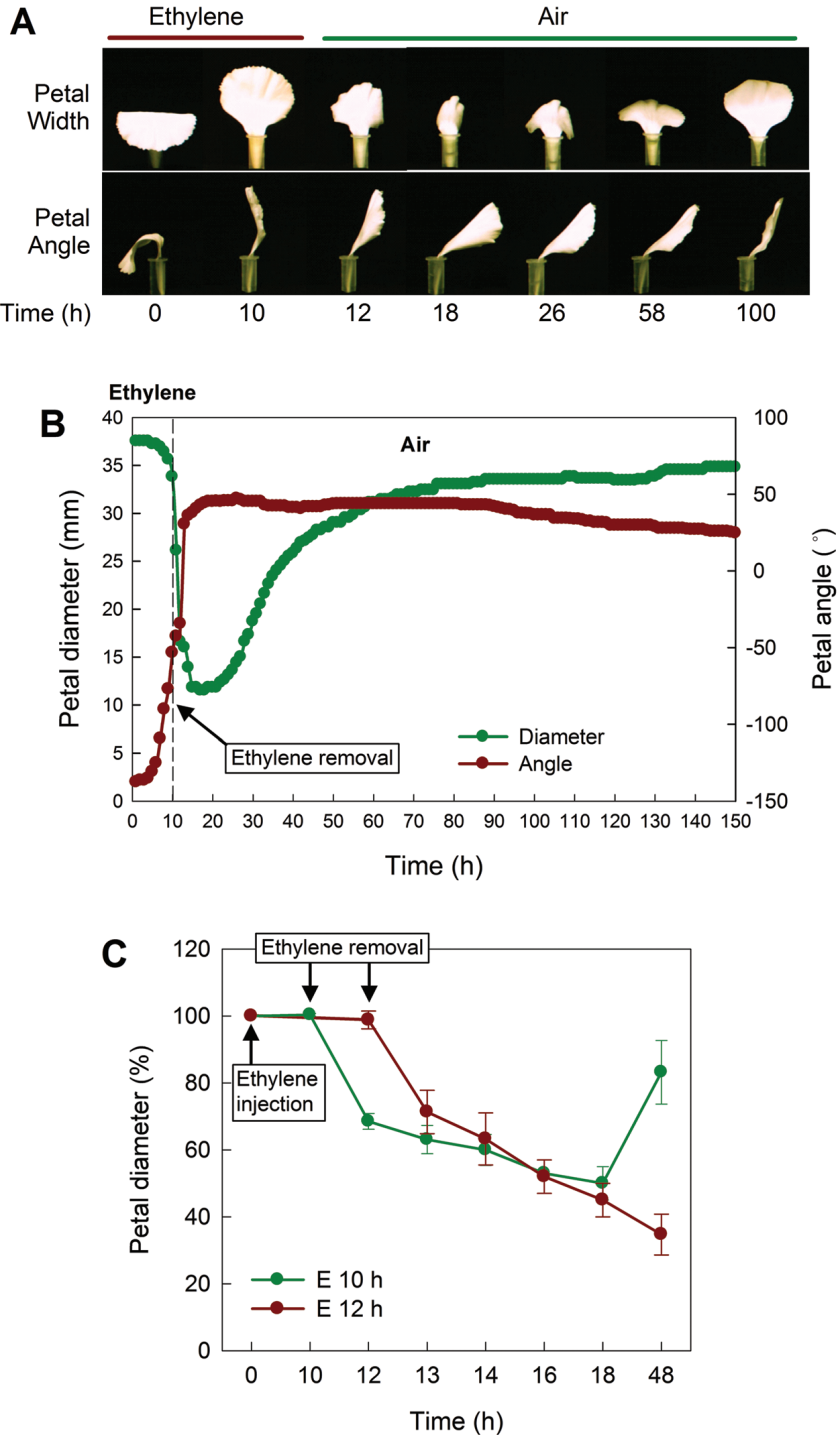
Inrolling and recovery patterns of petals. Petals detached from fully open flowers were held in micro tubes containing distilled water and treated with 10 µl l^–1^ ethylene for 10h. (A) Changes in petal inrolling and angle were photographed; and (B) petal diameter and angle were analysed from the images. Petal angle was defined as 0 ° when the petal stands vertically, –90 ° when the petal is perpendicular to 0 ° in an anticlockwise direction, and 90 ° when the petal is perpendicular to 0 ° in a clockwise direction. (C) Petals were incubated in the treatment chamber with 10 µl l^–1^ ethylene and the ethylene gas was removed from the chamber after 10h and 12h, respectively. Petal diameter was measured before ethylene treatment (0h), immediately after ethylene removal (10h and 12h), and at 13, 14, 16, 18, and 48h after ethylene removal. Data represent the mean ±SE of five replicates.

To determine recovery patterns associated with petal inrolling, petals were exposed to 10 µl l^–1^ ethylene for 10h, and the changes in petal diameter and angle were measured. The kinetic analysis revealed that petals have three phases of development associated with ethylene responses: phase 1, delay in response; phase 2, rapid inrolling; and phase 3, recovery (Fig. 2A, B). Prior to ethylene exposure, petal diameter and angle are ~38mm and –130 °, respectively. In phase 1, petal diameter changed minimally with ethylene exposure. In phase 2, the petals rapidly inrolled after the withdrawal of ethylene from the treatment chamber, losing ~70% of their initial diameter (11mm) within 8h; thus displaying complete inrolling of the petals. In phase 3, recovery from the inrolling was initiated 8h after ethylene removal and petals recovered their initial diameter within 48h.

Interestingly, the petal angle displayed changes earlier than inrolling in the presence of ethylene and rapidly changed vertically following the removal of ethylene. Petal angle changed ~190 ° from the initial angle (–130 °) within 8h, and rarely recovered with time. Since no correlation was seen with ethylene production, it was hypothesized that the change in petal angle is most probably related to floral opening, and that this developmental process is irreversible in carnation petals.

In order to determine whether the rapid response in petal inrolling results from the transition to air, petals were treated with ethylene and transferred to air after 10h and 12h treatment, respectively (Fig. 2C). While petal diameter was not changed during ethylene treatment, it rapidly decreased after ethylene removal after 10h and 12h of ethylene treatment. The petals that were treated with ethylene for 10h lost 40% of their initial diameter at 2h after ethylene removal (12h) and recovered after 48h. The petals treated with ethylene for 12h retained their initial diameter at 12h, but then their diameter continued to decrease quickly and did not recover. This result shows that the petal inrolling (diameter) response is initially delayed in the presence of ethylene and occurs immediately after the removal of exogenous ethylene.

### Relationship between petal inrolling and ethylene sensitivity

**Fig. 3.**
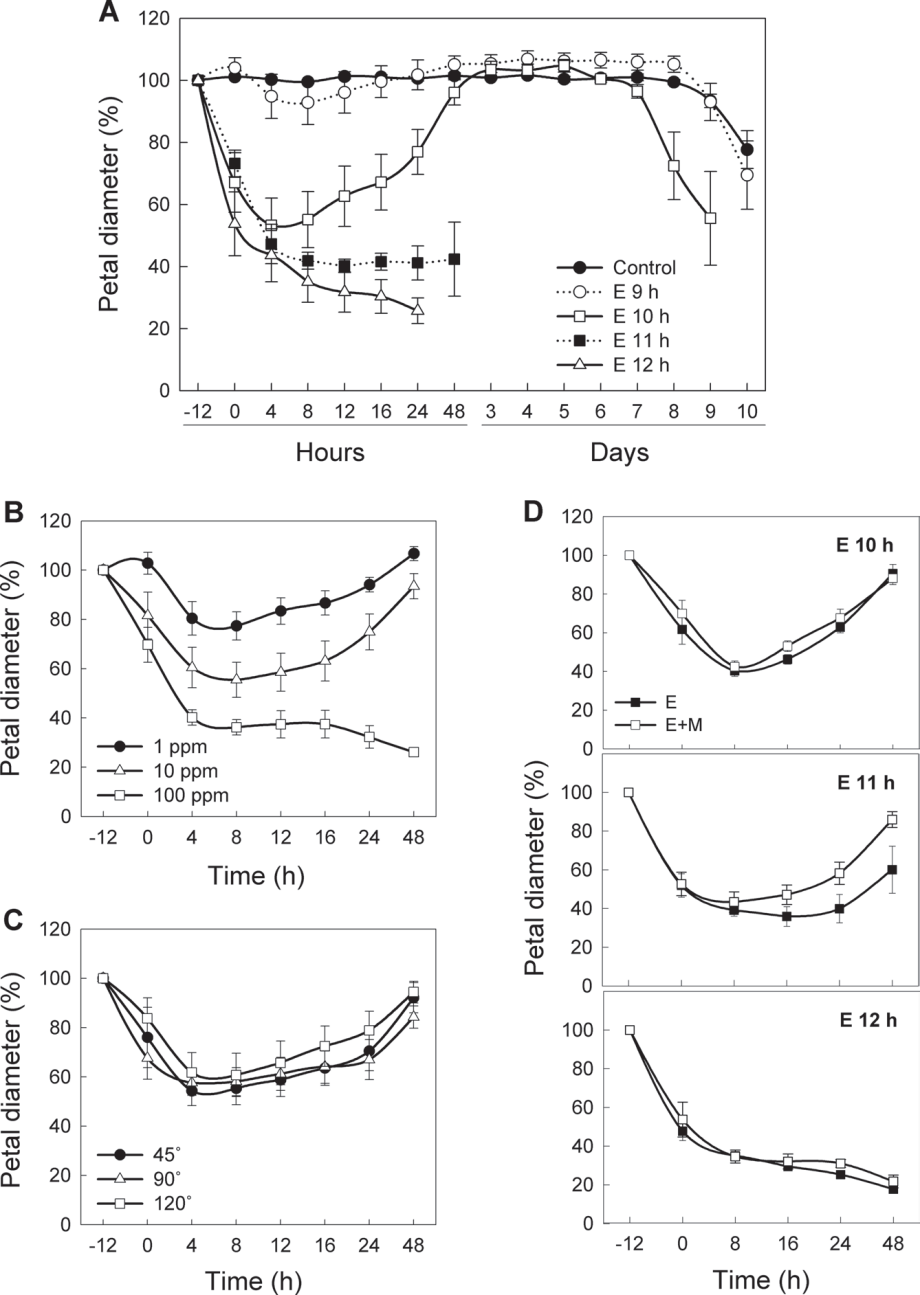
Change in petal diameter in response to ethylene and 1-MCP. Petals were (A) treated with 10 µl l^–1^ ethylene (E) for various times (9–12h) and (B) treated with various concentration of ethylene (1, 10, or 100 µl l^–1^) for 10h. (C) Petals in three different stages (45, 90, and 120 °) were treated with 10 µl l^–1^ ethylene for 10h. (D) Effect of 1-MCP on change in petal diameter. Petals were treated with 1-MCP (E+M) for 8h following ethylene treatment (E) for either 10, 11, or 12h. The control is air-treated petals. Petal diameter was measured before ethylene treatment (–12h) and at 0 (after 20min in air), 4, 8, 12, 16, 24, and 48h after ethylene removal. Data represent the mean ±SE of five replicates.

To determine the threshold time of ethylene perception for petal inrolling, petals were treated with 10 µl l^–1^ ethylene for 9, 10, 11, and 12h and transferred to air atmosphere at time 0. As shown in Fig. 3A, the petals treated for 9h with ethylene showed a slight decrease in petal diameter following the transfer to air. The petals treated for 10h with ethylene lost ~50% of their initial diameter at 8h, indicating a complete inrolling. However, the petals began to recover from the inrolling following the transfer to air and reverted to their initial diameter 40h later. On the other hand, the petals treated with ethylene for 11h and 12h fully inrolled at 8h and showed a complete senescence of petals due to inrolling, wilting, and browning within 16h (Fig. 3A).

The concentration of ethylene also significantly affected petal inrolling (Fig. 3B), and, following ethylene treatments, petals lost ~20, 40, and 65% of their initial diameter within 8h after treatment with 1, 10, and 100 µl l^–1^ ethylene, respectively. While the petals in 1 µl l^–1^ and 10 µl l^–1^ ethylene returned to their initial diameter within 48h after removal from ethylene, the petals in 100 µl l^–1^ ethylene failed to recover. On the other hand, there were no significant differences in petal diameter between the petals at different developmental stages. All petals showed similar inrolling at 8h, followed by the recovery from the inrolling (Fig. 3B). These results indicate that the recovery from petal inrolling depends on the duration and concentration of ethylene treatment rather than the stage of floral development, and that ethylene treatment lasting longer than the threshold time (12h) is required to elicit the sustained progression of petal senescence.

To demonstrate whether the progression of petal inrolling is caused by ethylene perception, carnation petals were treated with 1-MCP, an inhibitor of ethylene binding that has been shown to bind competitively to the ethylene receptors with a much higher affinity for the receptors than that of ethylene (Sisler *et al.*, 1996; Hall *et al.*, 2000; Binder *et al.*, 2004). Petals were treated with ethylene for 10, 11, and 12h and subsequently treated with 1-MCP. 1-MCP treatment following ethylene exposure slightly suppressed petal inrolling in the petals treated with ethylene for 11h, but was not able to disrupt the progress of petal inrolling in the petals treated with ethylene for 12h (Fig. 3D). This suggests that once the petal tissues have entered the climacteric phase, blocking of ethylene is not able to suppress the progression of petal senescence in carnations.

### Changes in the mRNA levels of ethylene biosynthesis genes during petal inrolling and recovery

**Fig. 4.**
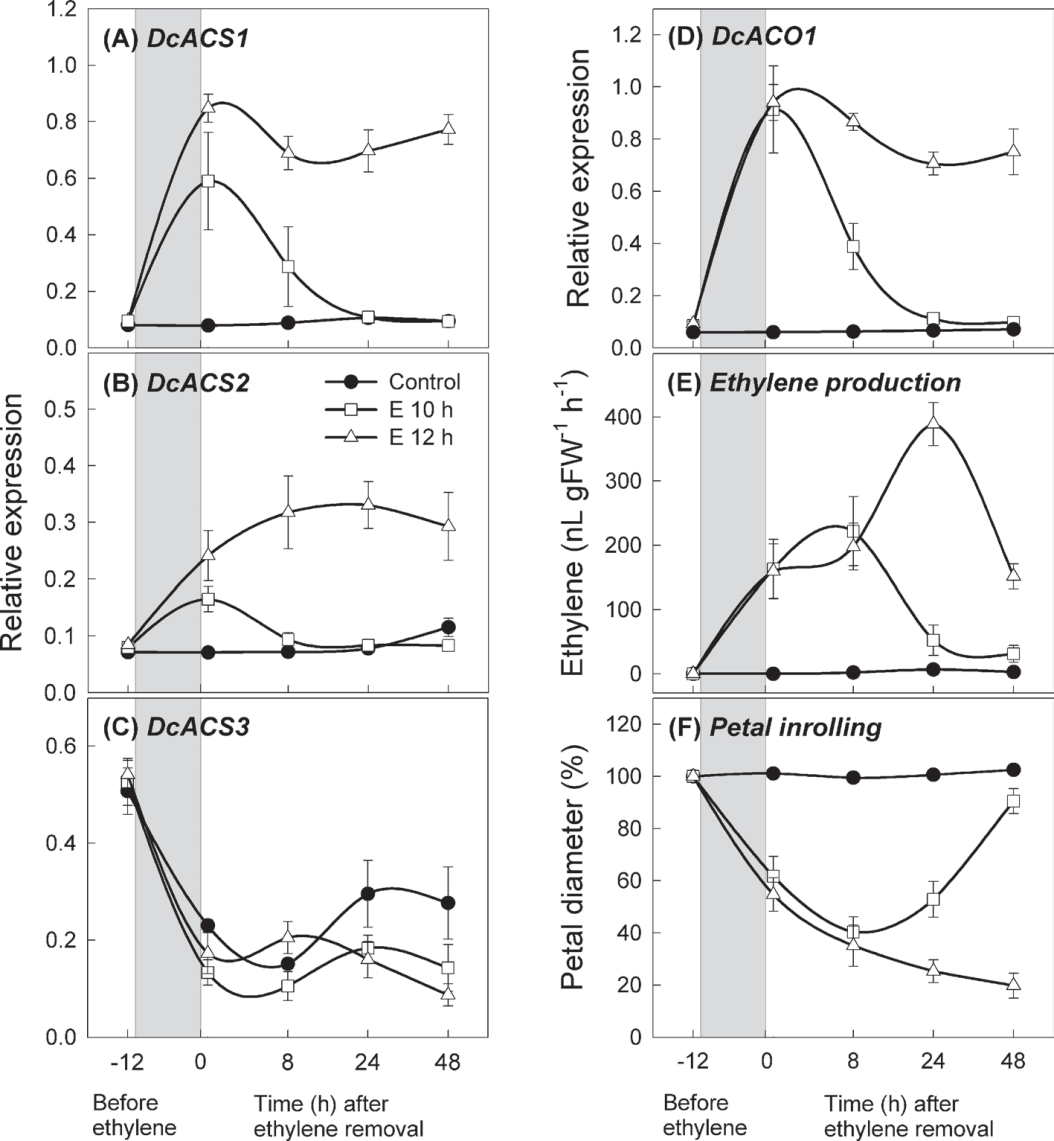
Expression of ethylene biosynthesis genes. (A) *DcACS1*, (B) *DcACS2*, (C) *DcACS3*, and (D) *DcACO1* in petals. (E) Ethylene production by petals. (F) Petal diameter. Petals were treated with air only (control), or 10 µl l^–1^ ethylene for 10h (E 10h) or 12h (E 12h). The grey boxes indicate the time period when the petals were exposed to ethylene. Petal samples were collected before ethylene treatment (–12h) and 20min, 8, 24, and 48h after ethylene removal. Data represent the mean ±SE of five replicates.

To determine whether the transcript levels of ethylene biosynthesis genes differed between petals that were inrolled or had recovered, RT–PCR analysis was conducted on petals treated with 10 µl l^–1^ ethylene for 10h (E10-petals) or 12h (E12-petals). While *DcACS1* transcript was undetectable in control petals, it greatly increased subsequent to ethylene treatment (Fig. 4A). After transfer of the petals to ethylene-free air, *DcACS1* gene transcript in E10-petals decreased rapidly and was undetectable at 24h. In contrast, the *DcACS1* transcript remained high in E12-petals for at least 48h. Although *DcACS2* generally showed lower expression than *DcACS1*, the expression pattern of *DcACS2* was similar to that of *DcACS1* in air and ethylene (Fig. 4B). On the other hand, *DcACS3* transcript levels were initially high, but declined for the first 12h. This occurred whether or not exogenous ethylene was applied and might be due to wounding and subsequent low levels of ethylene (Fig. 4C). In summary, differential regulation of the three ACS genes was observed in carnation during the senescence programme.

Additional biosynthesis genes were analysed to determine if changes were associated with ethylene treatment. *DcACO1* expression mirrored the expression patterns of *DcACS1*. Specifically, after ethylene treatment and subsequent transfer to air, *DcACO1* levels in E10-petals quickly decreased, while levels in E12-petals remained high (Fig. 4D). mRNA levels of *DcSAMS1* (SAM synthetase) were also measured and, while transcript was abundant in control petal tissues, it declined rapidly upon ethylene treatment. *DcSAMS1* expression returned to pre-treatment levels in E10-petals, but not in E12-petals (Supplementary Fig. S1 available at *JXB* online). However, overall ethylene production increased dramatically subsequent to ethylene treatment, and in E10-petals peaked at 8h before declining; whereas in E12-petals ethylene production peaked at 8h and was followed by a second dramatic increase at 24h (Fig. 4E). The second peak in ethylene synthesis was 2-fold higher than the first peak, and it was hypothesized that this represents the transition to climacteric phase. This parallels the morphological observations as E12-petals showed a steady decrease in petal diameter and a complete wilting at 24h associated with this second ethylene peak. In contrast, E10-petals showed a recovery in diameter at 24h simultaneously with the drop in ethylene synthesis (Fig. 4E, F).

The results show that the transcript levels of *DcACS1*, *DcACS2*, and *DcACO1* have similar patterns of regulation by ethylene and that changes in these genes generally correlate with changes in ethylene biosynthesis. When transcript levels decrease, ethylene levels decrease and petal recovery can occur. In contrast, when the levels of these genes remain elevated, ethylene levels rise and petal senescence occurs.

### Changes in the mRNA levels of ethylene receptors and downstream regulators during petal inrolling and recovery

**Fig. 5.**
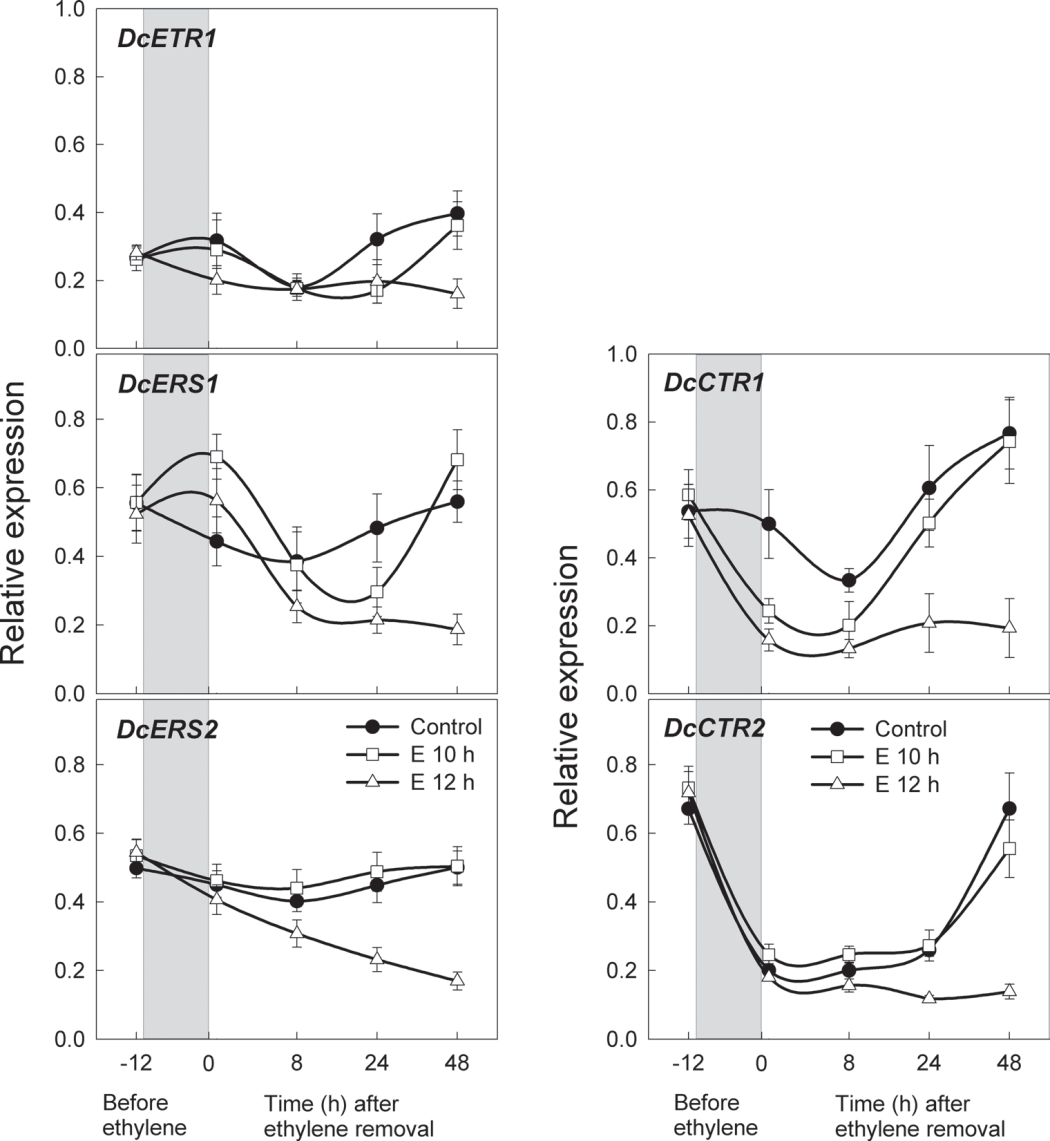
Expression of ethylene receptor genes, *DcETR1*, *DcERS1*, and *DCERS2,* and the downstream genes of the receptors, *DcCTR1* and *DcCTR2*, in petals. Petals were treated with air only (control), or 10 µl l^–1^ ethylene for 10h (E 10h) or 12h (E 12h). The grey boxes indicate the time period when the petals were exposed to ethylene. Petal samples were collected before ethylene treatment (–12h) and 20min, 8, 24, and 48h after ethylene removal. Data represent the mean ±SE of five replicates.

To identify the role of ethylene perception in regulation of petal inrolling and recovery, the transcript abundance of the ethylene receptors (*DcETR1*, *DcERS1*, and *DcERS2*), *CTR* genes, and *EIL* genes was determined in petals using RT–PCR. Three previously identified ethylene receptor genes, *DcETR1*, *DcERS1*, and *DcERS2* (Charng *et al.*, 1997; Shibuya *et al.*, 1998; Nagata *et al.*, 2000) were analysed. While transcript levels of these receptor genes showed some variation in control petals during the time-course of these experiments, these changes were distinct from the 12h treatment and are likely to be due to wounding. After ethylene treatment for 12h, gene expression of *DcETR1*, *DcERS1*, and *DcERS2* decreased. In contrast, 10h ethylene treatment resulted in transient changes in *DcETR1* and *DcERS1* transcripts and no change in *DcERS2* (Fig. 5).

**Fig. 6.**
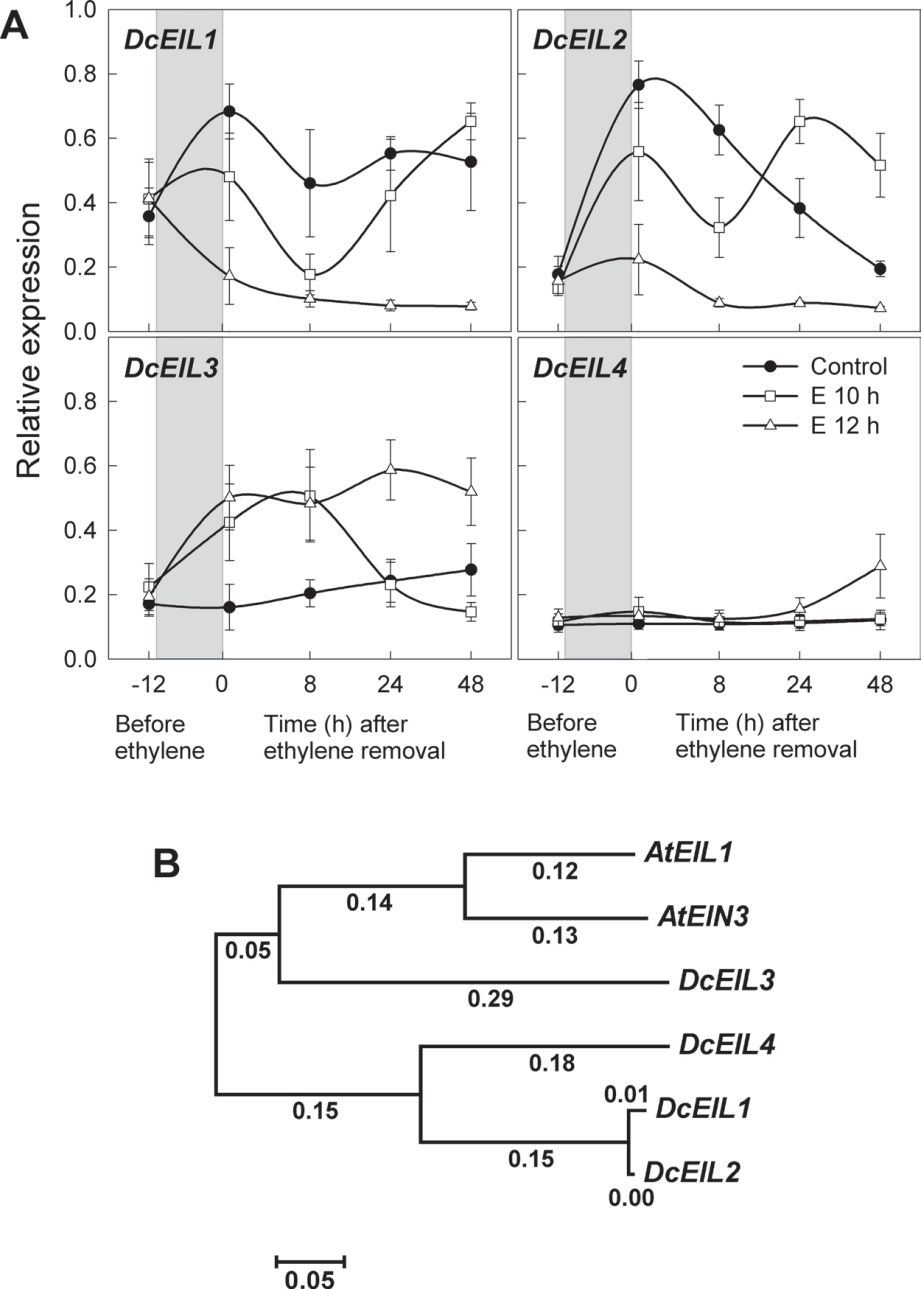
Expression of the transcription factors, *DcEIL1*, *DcEIL2* (*DcEIL1/2*), *DcEIL3*, and *DcEIL4* in petals. (A) Relative expression patterns. Petals were treated with air only (control), or 10 µl l^–1^ ethylene for 10h (E 10h) or 12h (E 12h). The grey boxes indicate the time period when the petals were exposed to ethylene. Petal samples were collected before ethylene treatment (–12h) and 20min, 8, 24, and 48h after ethylene removal. (B) Phylogenetic analysis. Protein sequences for the carnation EIL family (*DcEIL1–DcEIL4*), and *Arabidopsis EIN3* (*AtEIN3*) and *EIL1* (*AtEIL1*) were aligned and a phylogenetic tree was constructed by an unrooted Neighbor–Joining method based on the protein sequence similarities. Data represent the mean ±SE of five replicates.

Downstream targets of the receptors were also analysed including CTR, that functions as a negative regulator of ethylene signalling by forming a complex with the receptors on the endoplasmic reticulum (Kieber *et al.*, 1993; Gao *et al.*, 2003; Huang *et al.*, 2003), and *EIN3*-Like (EIL) transcription factors predicted to function downstream of *CTR1*. Application of ethylene for 10h caused a transient decrease in the transcript levels of both *DcCTR1* and *DcCTR2*, and 12h treatment with ethylene resulted in a prolonged decrease in the levels of both genes (Fig. 5). These decreases in *CTR* gene levels are predicted to cause constitutive ethylene signalling for as long as *CTR* gene level are low. The four *EIL* transcription factor genes (*DcEIL1–DcEIL4*) identified in carnations (Waki *et al.*, 2001; Iordachescu and Verlinden, 2005) were also analysed. While *DcEIL1* and *DcEIL2* (*DcEIL1/2*) showed relatively inconsistent expression patterns (Fig. 6A), transcript levels of *DcEIL3* increased considerably with ethylene treatment. Interestingly, 24h after ethylene removal, *DcEIL3* transcript in E10-petals rapidly dropped to levels similar to those in untreated petals; however, *DcEIL3* levels in E12-petals remained high for 48h. *DcEIL4* transcript increased only slightly in E12-petals at 48h. Increased levels of *DcEIL3* have been predicted to lead to ethylene responses (Iordachescu and Verlinden, 2005), and the present results support previous observations.

Since *EIL1* is phylogenetically and functionally closely related to *EIN3*, a positive regulator of ethylene perception (Binder *et al.*, 2007), the relationship among *EIL* and *EIN3* family members in carnation and *Arabidopsis* was examined. A phylogenetic analysis (Neighbor–Joining method) was performed based on predicted protein sequences, and it was determined that *DcEIL3* is more closely related to *Arabidobsis EIN3* and *EIL1* than the other *DcEIL* genes (Fig. 6B). It is hypothesized that *DcEIL3* contributes most to ethylene signalling, consistent with the observation that only *DcEIL3* showed an expression pattern that changed in response to ethylene. These results are consistent with previous work showing that *DcEIL3* is regulated by ethylene and during flower senescence (Iordachescu and Verlinden, 2005).

### Treatment of petals with ethylene synthesis antagonists

**Fig. 7.**
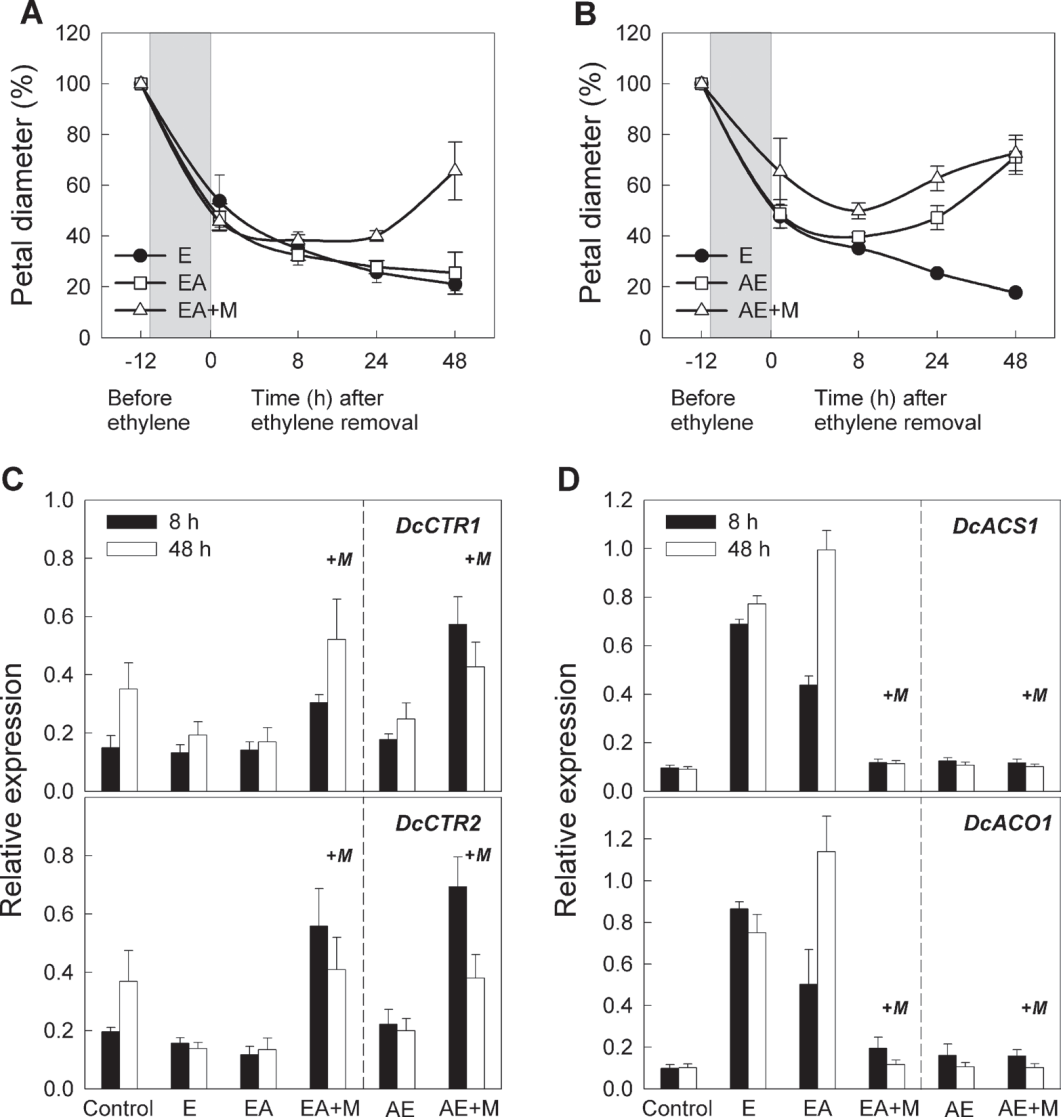
Effects of AVG and 1-MCP on changes in petal diameter and gene expression. (A) Petal diameter when the petals were treated simultaneously with 12h ethylene (E) and AVG (EA) and subsequently with 1-MCP (+M). (B) Petal diameter when the petals were treated with AVG prior to 12h ethylene (AE) and subsequently 1-MCP. Petals were kept in the AVG solution throughout the experiment once the treatment had started. The grey boxes indicate the time period when the petals were exposed to ethylene. Petal diameter was measured before ethylene treatment (–12h) and 20min, 8, 24, and 48 after ethylene removal. The relative expression of (C) *DcCTR1* and *DcCTR2* and (D) *DcACS1* and *DcACO1* was detected at 8h and 48h after ethylene removal. Data represent the mean ±SE of five replicates.

To confirm further that ACS may be a main determinant in the transition to climacteric phase, petals were treated with AVG, an inhibitor of ACS activity (Baker *et al.*, 1977; Yang and Hoffman, 1984). When petals were simultaneously treated with AVG and 12h ethylene (EA), AVG was not able to suppress petal inrolling. However, combined addition of AVG and 1-MCP with 12h ethylene treatment (EA+M) interrupted the inrolling progress, allowing recovery (Fig. 7A). Transcript levels of *DcCTR1* and *DcCTR2* were considerably lowered by 12h ethylene treatment (E), and addition of AVG (EA) was not able to inhibit this decrease in the transcript levels. Alternatively, EA+M effectively prevented the decrease in *DcCTR1* and *DcCTR2* transcripts at 8h and 48h (Fig. 7C). In addition, the ethylene-induced increases in *DcACS1* and *DcACO1* transcripts were not inhibited by AVG (EA), but were completely suppressed by EA+M (Fig. 7D). These results combined with results shown in Fig. 4 indicate that once ethylene sensitivity has reached a particular threshold level, it requires both 1-MCP to block ethylene perception and AVG to block ethylene synthesis in order to inhibit the progression of senescence.

In contrast, when the petals were treated with AVG prior to ethylene exposure (AE), AVG promoted recovery from inrolling at later stages. Treatment with 1-MCP (AE+M) effectively inhibited the inrolling earlier at 24h yet demonstrated similar phenotypic responses, as no differences were observed in the final petal diameter at 48h between AE and AE+M (Fig. 7B). In summary, AE did not inhibit the decrease in *DcCTR1* and *DcCTR2* in ethylene-treated petals, but suppressed the *DcACS1* and *DcACO1* transcripts at 8h and 48h. AE+M effectively inhibited the reduction in *DcCTR1* and *DcCTR2* transcripts and suppressed the ethylene-inducible increase in *DcACS1* and *DcACO1* transcripts (Fig. 7C, D).

**Fig. 8.**
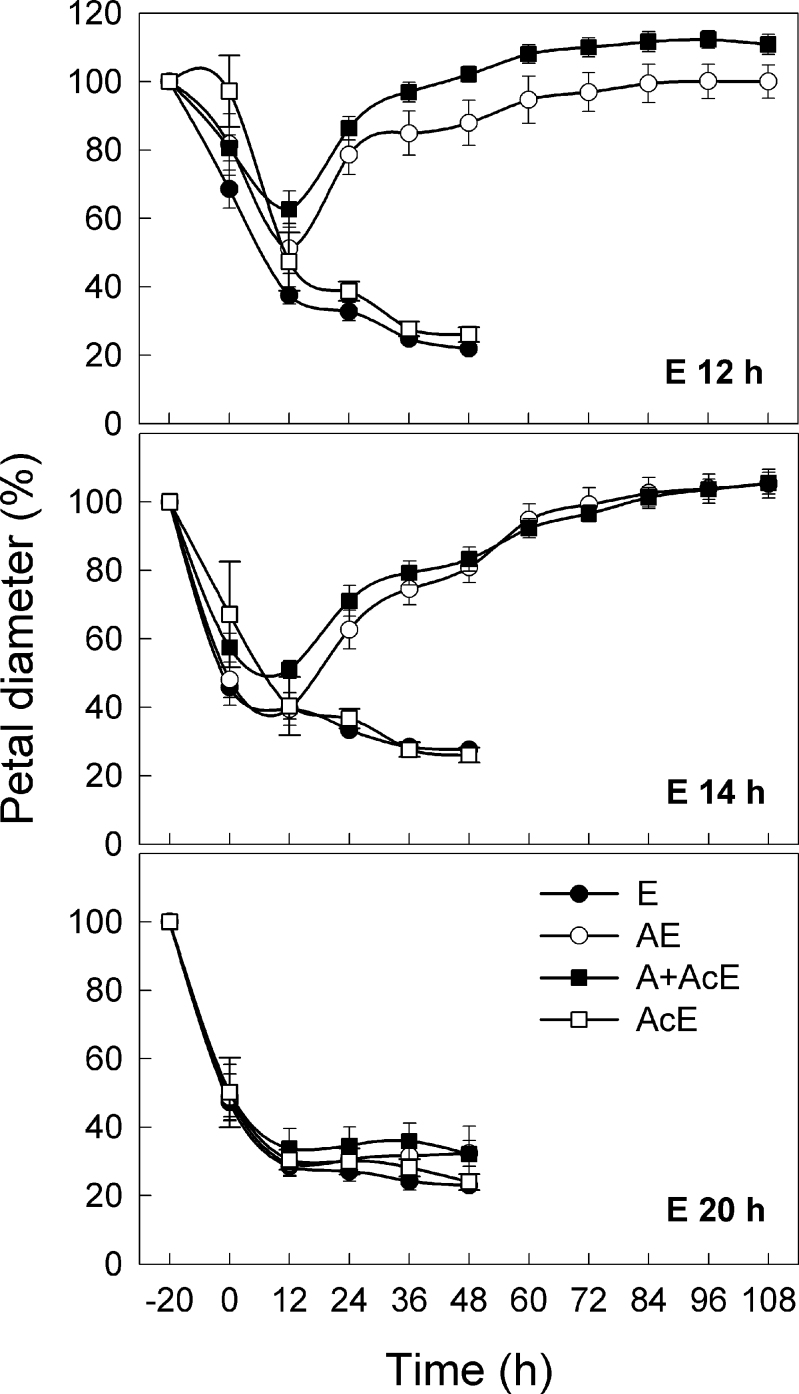
Effects of AVG and ACBC on change in petal diameter. Petals were treated with AVG (AE) or AVG+ACBC (A+AcE) before ethylene treatment (E) for either 12, 14, or 20h. Petals were kept in the AVG or AVG+ACBC solution throughout the experiment once the treatment had started. Petal diameter was measured before ethylene treatment (–20h) and at 0h (after 20min in air) and every 12h after ethylene removal. Data represents the mean ±SE of five replicates.

These data led to the hypothesis that levels of *DcCTR1* and *DcCTR2* are strongly correlated with *DcACS1* and *DcACO1* transcripts. These results also suggest that suppression of ACS is able to inhibit *DcACS1* and *DcACO1* transcripts independently of the inactivation of *DcCTR* genes. To determine whether the transition to climacteric phase by ethylene can be inhibited by suppression of ACS and ACO activity, petals were simultaneously treated with AVG and ACBC, an inhibitor of ACO activity (Kosugi *et al.*, 1997), for 12h prior to ethylene exposure. As shown in Fig. 8, petal diameter rapidly decreased by ethylene treatment (E) and the petal inrolling was not inhibited by ACBC single treatment (AcE). In contrast, inrolling induced by 12h or 14h ethylene treatment was interrupted by treatments with AVG (AE) or AVG plus ACBC (A+AcE). Petals treated with 12h ethylene completely recovered from the inrolling at 72h with AE and at 48h with A+AcE. Even when the petals were treated with ethylene for 14h, the petals attained their pre-treatment diameter by 72h with AE or A+AcE. These results indicate that ACS activity is essential for transition to the climacteric phase and that the suppression of ACS interrupts the senescence programme.

## Discussion

The first morphological indication of senescence via petal inrolling occurs subsequent to a surge of ethylene synthesis in the flowers at the climacteric phase. During senescence, petals roll inward as a consequence of asymmetric tissue sensitivity to ethylene, as continuous perception of ethylene results in lipid peroxidation, loss of membrane integrity on the adaxial side of petals, and a subsequent reduction in turgor pressure leading to shrinkage of cells (Thompson, 1988; Kim *et al.*, 1998).

While it has generally been thought that the inrolling of petals during natural flower senescence is an irreversible process (Halevy and Mayak, 1981), some previous studies suggested reversibility of senescence or hormone sensitivity in response to exogenous ethylene in plant tissues (Molisch, 1938; Woltering and Harkema, 1987; Woodson and Lawton, 1988; ten Have and Woltering, 1997; Thomas *et al.*, 2003). In this study, unique phases of development were identified and it was determined that this process can be reversible.

Kinetic analyses indicate that petals treated for 10h with ethylene demonstrate three distinct phases in ethylene responses: phase 1, an initial lag phase during ethylene treatment before morphological changes become evident; phase 2, rapid inrolling of petals after removal from ethylene; and phase 3, the recovery and/or steady state. Interestingly, the petals rarely responded to ethylene in the presence of the hormone (phase 1) and rapidly lost ~50% of their initial diameter within 2h after the withdrawal of ethylene from the treatment chamber (phase 2). It is hypothesized that the delay in response (phase 1) and rapid transition from phase 1 to 2 might be caused by an inhibitory effect of exogenous ethylene (autoinhibition) resulting from suppression of ACC synthesis and ACS activity (Riov and Yang, 1982; Liu *et al.*, 1985; Philosophhadas *et al.*, 1985; Kende, 1993). In this model, after ethylene removal, ACC becomes available and ACS is active, releasing autoinhibition. Potentially, this leads to an increase in ethylene synthesis, concomitant with rapid inrolling of petals. This hypothesis is supported by previous studies demonstrating that application of exogenous ACC or inhibition of ethylene binding restores ethylene production in the tissues under autoinhibition (Riov and Yang, 1982; Philosophhadas *et al.*, 1985). Once the tissues reach the climacteric phase, exogenous ethylene is no longer inhibitory, as shown in the result of 20h ethylene treatment in Fig. 8. The present findings combined with previous work on tomato suggest unique roles for the different ACS members within specific tissues during the pre-climacteric and climacteric phase (Barry *et al.*, 2000; Barry and Giovannoni, 2007; Tsuchisaka *et al.*, 2009).

It was observed that recovery from inrolling coincided with increases in receptor transcript levels. This led to the hypothess that both receptors that have lost ethylene due to dissociation as well as the newly synthesized, ethylene-free receptors are active and repressing ethylene signalling. For example, ethylene dissociates with a half-life of 10–12h from the *AtETR1* and *AtETR2* receptors, with nearly all the ethylene released after 36h (Schaller and Bleecker, 1995; O’Malley *et al.*, 2005; McDaniel and Binder, 2012). This timing approximates the recovery pattern of the petals (initial signs of recovery 8h after ethylene removal and full recovery 40h later). Thus, recovery from petal inrolling is strongly correlated with the reversion of the receptors to their initial active state, after the dissociation of ethylene.

The results also imply that the suppression of *DcACS3* by ethylene is coincident with the activation of *DcACS2*, and that the accumulation of *DcACS2* during the transition period is found together with the sustained accumulation of *DcACS1* transcripts, resulting in the initiation of the autocatalytic ethylene production and senescence of petals. This hypothesis is supported by the fact that at the transition point (8h), low *DcACS3* levels and high *DcACS2* levels were observed, followed by an increase in the amount of *DcACS1* in the E12-petals (Fig. 4A–C). There is precedence for this differential regulation of ACS family members in a tissue-specific manner during development, senescence, and fruit ripening (Kende, 1993; Jones and Woodson, 1999*a*; Barry and Giovannoni, 2007; Tsuchisaka *et al.*, 2009). For example, in tomato plants, *LeACS1A* and *LeACS6* are responsible for system 1 ethylene synthesis, but *LeACS4* and *LeACS2* function during the transition period and fruit ripening, respectively (Barry *et al.*, 2000; Barry and Giovannoni, 2007). It is hypothesized that *DcACS3* could be responsible for system 1 ethylene synthesis which functions during flower development.

Gene expression in response to ethylene has revealed that there is an inverse relationship between the transcript levels of ethylene receptor genes versus *DcACS1* and *DcACO1*. This result supports the previous study that demonstrated a decrease in *DcERS2* transcript and a simultaneous increase in ethylene synthesis during senescence of carnation flowers (Shibuya *et al.*, 2002). The decrease in *DcETR1*, *DcERS1*, and *DcERS2* transcripts was correlated with the reduction in *DcCTR1* and *DcCTR2* transcripts, and also the induction of the transcription factor *DcEIL3*. These results are consistent with the model that the receptors are negative regulators of ethylene signalling (Hua and Meyerowitz, 1998; Tieman *et al.*, 2000). Ethylene binding by the receptors suppresses *CTR1* action immediately downstream of the receptors, thereby switching on downstream signalling events, resulting in the activation of the positive regulator *EIN2* and the subsequent induction of the transcription factor *EIN3* (Chao *et al.*, 1997; Alonso *et al.*, 1999; Yanagisawa *et al.*, 2003). In accordance with these interpretations, it is propose that the disappearance of the *DcCTR* transcripts following 12h ethylene treatment may represent the turning-off state of CTR action, and this ultimately induces the accumulation of transcription factor *DcEIL3*, resulting in sustaining of the petal senescence process.

Despite the identification of many key components in ethylene signal transduction, there are still many unanswered questions as to how ethylene signalling is connected to ethylene biosynthesis. It is interesting to note that when *DcCTR1* transcript in E10-petals began to recover 24h after transfer to air, *DcEIL3* was completely suppressed simultaneously with the disappearance of *DcACS1* and *DcACO1*. Considering these results, it is believed that this supports the idea that levels of DcCTRs and DcEIL3 contribute to the changes in sensitivity to ethylene in carnation petals.

Mitogen-activated protein kinase (MAPK) modules are also involved in regulating the ethylene biosynthesis pathway. While it is still unclear how the MAPK cascade module is linked to the ethylene signal response pathway, the present data support that *DcCTR* transcripts are inversely correlated to *DcACS1* and *DcACO1* transcripts. When ethylene binds to the receptor–CTR1 complex, *CTR1* is inactivated; thus no longer blocking downstream signalling events. MPK modules such as MPK3/6 appear to be involved in regulating ethylene biosynthesis by increasing the accumulation of some ACS members; however, the MPK modules are suppressed by *CTR1* activation (Ouaked *et al.*, 2003; Christians *et al.*, 2009; Hahn and Harter, 2009). Our work on carnation shows that the accumulation of *DcACS1* and *DcACO1* is a strict requirement for the transition to climacteric phase and, once the autocatalysis has initiated, *DcACS1* and *DCACO1* levels remained high. Whether *DcCTR1* levels might influence the expression of these two enzymes is unknown. The results demonstrated that inhibition of ACS prior to ethylene exposure was not able to prevent reduction in transcripts of *DcCTR* genes, yet inhibited transcripts of *DcACS1* and subsequently *DcACO1*. This is also supported by work in *Arabidopsis* in which the ethylene-constitutive mutant *ctr1* has higher levels of *ACO2* and produces higher levels of ethylene than the wild type subsequent to ACC application (van Zhong and Burns, 2003; Liu and Zhang, 2004). In the future, the relationship between *DcCTR* genes and *DcACS1* could be more clearly understood by protein analysis.

In carnation flowers, ACC appears to accumulate sequentially within floral organs styles, ovaries, receptacles, and the lower and upper portions of petals (Bufler *et al.*, 1980; Nichols *et al.*, 1983; Mor *et al.*, 1985; Jones and Woodson, 1999*b*; Shibuya *et al.*, 2000). The translocation of ACC from the lower to the upper portion of petals is the essential requirement for an increase in ethylene sensitivity, and ethylene synthesis in the petals is associated with a corresponding increase in the endogenous ACC level (Mor *et al.*, 1985; Wang and Woodson, 1989; Overbeek and Woltering, 1990; ten Have and Woltering, 1997). It has also been shown that in carnation flowers, ethylene synthesis is suppressed by the removal of gynoecium, which is a main site of ethylene synthesis, and restored following ACC application (Shibuya *et al.*, 2000). Furthermore, ACC treatment enhances ACS and ACO transcripts in the style tissues under blocking of ethylene binding (Jones, 2003). The implication from these results is that ACC (or perhaps ACS itself) is strongly related to both changes in ethylene sensitivity and ethylene signalling. This is supported by previous studies that showed that a drop in the receptor levels coincides with autocatalytic ethylene synthesis, which is mediated by specific members of the ACS family (Riov and Yang, 1982; Barry and Giovannoni, 2007; Kevany *et al.*, 2007).

In summary, it is hypothesized that *DcACS1* acts as a signalling molecule during senescence and is correlated with the decrease of *DcCTR* genes and the increase of *DcEIL3*, indicating that petal tissues have entered the climacteric phase and that senescence proceeds irreversibly. Importantly, it has been shown that senescence progression of petals can be interrupted when *DcACS1* is suppressed prior to complete senescence in carnation petals.

## Supplementary data

Supplementary data are available at *JXB* online.

Figure S1. Expression of *DcSAMS1* in petals.

Supplementary Data

## References

[CIT0001] Alonso JM Hirayama T Roman G Nourizadeh S Ecker JR 1999 EIN2, a bifunctional transducer of ethylene and stress responses in Arabidopsis. Science 284, 2148–2152 10381874 10.1126/science.284.5423.2148

[CIT0002] Baker JE Wang CY Lieberman M Hardenburg R 1977 Delay of senescence in carnations by a rhizobitoxine analog and sodium benzoate. Hortscience 12, 38–39

[CIT0003] Barry CS Giovannoni JJ 2007 Ethylene and fruit ripening. Journal of Plant Growth Regulation 26, 143–159

[CIT0004] Barry CS Llop-Tous MI Grierson D 2000 The regulation of 1-aminocyclopropane-1-carboxylic acid synthase gene expression during the transition from system-1 to system-2 ethylene synthesis in tomato. Plant Physiology 123, 979–986 10889246 10.1104/pp.123.3.979 PMC59060

[CIT0005] Binder BM Mortimore LA Stepanova AN Ecker JR Bleecker AB 2004 Short-term growth responses to ethylene in Arabidopsis seedlings are EIN3/EIL1 independent. Plant Physiology 136, 2921–2927 15466219 10.1104/pp.104.050393 PMC523354

[CIT0006] Binder BM Walker JM Gagne JM Emborg TJ Hemmann G Bleecker AB Vierstra RD 2007 The Arabidopsis EIN3 binding F-box proteins EBF1 and EBF2 have distinct but overlapping roles in ethylene signaling. The Plant Cell 19, 509–523 17307926 10.1105/tpc.106.048140 PMC1867343

[CIT0007] Borochov A Woodson WR 1989 Physiology and biochemistry of flower petal senescence. Horticultural Reviews 11, 15–43

[CIT0008] Bufler G Mor Y Reid MS Yang SF 1980 Changes in 1-aminocyclopropane-1-carboxylic acid content of cut carnation flowers in relation to their senescence. Planta 150, 439–442 10.1007/BF00390183 24306897

[CIT0009] Chao QM Rothenberg M Solano R Roman G Terzaghi W Ecker JR 1997 Activation of the ethylene gas response pathway in Arabidopsis by the nuclear protein ETHYLENE-INSENSITIVE3 and related proteins. Cell 89, 1133–1144 9215635 10.1016/s0092-8674(00)80300-1

[CIT0010] Charng YY Sun CW Yan SL Chou SJ Chen YR Yang SF 1997 cDNA sequence of a putative ethylene receptor from carnation petals. Plant Physiology 115, 8639411456

[CIT0011] Christians MJ Gingerich DJ Hansen M Binder BM Kieber JJ Vierstra RD 2009 The BTB ubiquitin ligases ETO1, EOL1 and EOL2 act collectively to regulate ethylene biosynthesis in Arabidopsis by controlling type-2 ACC synthase levels. The Plant Journal 57, 332–345 18808454 10.1111/j.1365-313X.2008.03693.x PMC2807402

[CIT0012] Gao ZY Chen YF Randlett MD Zhao XC Findell JL Kieber JJ Schaller GE 2003 Localization of the Raf-like kinase CTR1 to the endoplasmic reticulum of Arabidopsis through participation in ethylene receptor signaling complexes. Journal of Biological Chemistry 278, 34725–34732 12821658 10.1074/jbc.M305548200

[CIT0013] Hahn A Harter K 2009 Mitogen-activated protein kinase cascades and ethylene: signaling, biosynthesis, or both? Plant Physiology 149, 1207–1210 19109412 10.1104/pp.108.132241 PMC2649397

[CIT0014] Halevy AH Mayak S 1981 Senescence and postharvest physiology of cut flowers. Part 2. Horticultural Reviews 3, 59–143

[CIT0015] Hall AE Findell JL Schaller GE Sisler EC Bleecker AB 2000 Ethylene perception by the ERS1 protein in Arabidopsis. Plant Physiology 123, 1449–1457 10938361 10.1104/pp.123.4.1449 PMC59101

[CIT0016] Hua J Meyerowitz EM 1998 Ethylene responses are negatively regulated by a receptor gene family in Arabidopsis thaliana. Cell 94, 261–271 9695954 10.1016/s0092-8674(00)81425-7

[CIT0017] Huang Y Li H Hutchison CE Laskey J Kieber JJ 2003 Biochemical and functional analysis of CTR1, a protein kinase that negatively regulates ethylene signaling in Arabidopsis. The Plant Journal 33, 221–233 12535337 10.1046/j.1365-313x.2003.01620.x

[CIT0018] Iordachescu M Verlinden S 2005 Transcriptional regulation of three EIN3-like genes of carnation (*Dianthus caryophyllus* L. cv. Improved White Sim) during flower development and upon wounding, pollination, and ethylene exposure. Journal of Experimental Botany 56, 2011–2018 15983019 10.1093/jxb/eri199

[CIT0019] Jones ML 2003 Ethylene biosynthetic genes are differentially regulated by ethylene and ACC in carnation styles. Plant Growth Regulation 40, 129–138

[CIT0020] Jones ML Woodson WR 1999a Differential expression of three members of the 1-aminocyclopropane-1-carboxylate synthase gene family in carnation. Plant Physiology 119, 755–764 9952472 10.1104/pp.119.2.755 PMC32153

[CIT0021] Jones ML Woodson WR 1999b Interorgan signaling following pollination in carnations. Journal of the American Society for Horticultural Science 124, 598–604

[CIT0022] Kende H 1993 Ethylene biosynthesis. Annual Review of Plant Physiology and Plant Molecular Biology 44, 283–307

[CIT0023] Kevany BM Tieman DM Taylor MG Dal Cin V Klee HJ 2007 Ethylene receptor degradation controls the timing of ripening in tomato fruit. The Plant Journal 51, 458–467 17655616 10.1111/j.1365-313X.2007.03170.x

[CIT0024] Kieber JJ Rothenberg M Roman G Feldmann KA Ecker JR 1993 Ctr1, a negative regulator of the ethylene response pathway in arabidopsis, encodes a member of the Raf family of protein-kinases. Cell 72, 427–441 8431946 10.1016/0092-8674(93)90119-b

[CIT0025] Kim E Son K Lee S Oh S-E 1998 The inrolling phenomena of petals during senescence in cut carnations (*Dianthus caryophyllus* L. cv. Shinkibo). Journal of Plant Biology 41, 304–311

[CIT0026] Kosugi Y Oyamada N Satoh S Yoshioka T Onodera E Yamada Y 1997 Inhibition by 1-aminocyclobutane-1-carboxylate of the activity of 1-aminocyclopropane-1-carboxylate oxidase obtained from senescing petals of carnation (*Dianthus caryophyllus* L) flowers. Plant and Cell Physiology 38, 312–318 9150604 10.1093/oxfordjournals.pcp.a029168

[CIT0027] Liu Y Su LY Yang SF 1985 Ethylene promotes the capability to malonylate 1-aminocyclopropane-1-carboxylic acid and d-amino acids in preclimacteric tomato fruits. Plant Physiology 77, 891–895 16664157 10.1104/pp.77.4.891 PMC1064626

[CIT0028] Liu YD Zhang SQ 2004 Phosphorylation of 1-aminocyclopropane-1-carboxylic acid synthase by MPK6, a stress-responsive mitogen-activated protein kinase, induces ethylene biosynthesis in Arabidopsis. The Plant Cell 16, 3386–3399 15539472 10.1105/tpc.104.026609 PMC535880

[CIT0029] Mayak S Kofranek AM 1976 Altering sensitivity of carnation flowers (*Dianthus caryophyllus* L.) to ethylene. Journal of the American Society for Horticultural Science 101, 503–506

[CIT0030] McDaniel BK Binder BM 2012 Ethylene receptor 1 (ETR1) is sufficient and has the predominant role in mediating inhibition of ethylene responses by silver in *Arabidopsis thaliana* . Journal of Biological Chemistry 287, 26094–26103 22692214 10.1074/jbc.M112.383034 PMC3406693

[CIT0031] McMurchie EJ McGlasson WB Eaks IL 1972 Treatment of fruit with propylene gives information about the biogenesis of ethylene. Nature 237, 235–236 4557321 10.1038/237235a0

[CIT0032] Molisch H 1938 The longevity of plants . Lancaster, PA: Science Press

[CIT0033] Mor Y Halevy AH Spiegelstein H Mayak S 1985 The site of 1-aminocyclopropane-1-carboxylic acid synthesis in senescing carnation petals. Physiologia Plantarum 65, 196–202

[CIT0034] Nagata M Tanikawa N Onozaki T Mori H 2000 Ethylene receptor gene (*ETR*) homolog from carnation. Journal of the Japanese Society for Horticultural Science 69, Supplement 1, 407

[CIT0035] Nichols R Bufler G Mor Y Fujino DW Reid MS 1983 Changes in ethylene production and 1-aminocyclopropane-1-carboxylic acid content of pollinated carnation flowers. Journal of Plant Growth Regulation 2, 1–8

[CIT0036] O’Malley RC Rodriguez FI Esch JJ Binder BM O’Donnell P Klee HJ Bleecker AB 2005 Ethylene-binding activity, gene expression levels, and receptor system output for ethylene receptor family members from Arabidopsis and tomato. The Plant Journal 41, 651–659 15703053 10.1111/j.1365-313X.2004.02331.x

[CIT0037] Ouaked F Rozhon W Lecourieux D Hirt H 2003 A MAPK pathway mediates ethylene signaling in plants. EMBO Journal 22, 1282–1288 12628921 10.1093/emboj/cdg131 PMC151067

[CIT0038] Overbeek JHM Woltering EJ 1990 Synergistic effect of 1-aminocyclopropane-1-carboxylic acid and ethylene during senescence of isolated carnation petals. Physiologia Plantarum 79, 368–376

[CIT0039] Philosophhadas S Meir S Aharoni N 1985 Autoinhibition of ethylene production in tobacco leaf-disks—enhancement of 1-aminocyclopropane-1-carboxylic acid conjugation. Physiologia Plantarum 63, 431–437

[CIT0040] Reid MS Çelikel FG 2008 Use of 1-methylcyclopropene in ornamentals: carnations as a model system for understanding mode of action. Hortscience 43, 95–98

[CIT0041] Riov J Yang SF 1982 Autoinhibition of ethylene production in citrus peel disks—suppression of 1-aminocyclopropane-1-carboxylic acid synthesis. Plant Physiology 69, 687–690 16662276 10.1104/pp.69.3.687 PMC426281

[CIT0042] Schaller GE Bleecker AB 1995 Ethylene-binding sites generated in yeast expressing the Arabidopsis ETR1 gene. Science 270, 1809–1811 8525372 10.1126/science.270.5243.1809

[CIT0043] Schaller GE Kieber JJ 2002 Ethylene. In: Somerville CR Meyerowitz EM, eds. The Arabidopsis Book . Rockville, MD: American Society of Plant Biologists

[CIT0044] Shibuya K Nagata M Tanikawa N Yoshioka T Hashiba T Satoh S 2002 Comparison of mRNA levels of three ethylene receptors in senescing flowers of carnation (*Dianthus caryophyllus* L.). Journal of Experimental Botany 53, 399–406 11847237 10.1093/jexbot/53.368.399

[CIT0045] Shibuya K Satoh S Yoshioka T 1998 A cDNA endoding a putative ethylene receptor related to petal senescence in carnation (*Dianthus caryophyllus* L.) flowers. Plant Physiology 116, 867

[CIT0046] Shibuya K Yoshioka T Hashiba T Satoh S 2000 Role of the gynoecium in natural senescence of carnation (*Dianthus caryophyllus* L.) flowers. Journal of Experimental Botany 51, 2067–2073 11141180 10.1093/jexbot/51.353.2067

[CIT0047] Sisler EC Dupille E Serek M 1996 Effect of 1-methylcyclopropene and methylenecyclopropane on ethylene binding and ethylene action on cut carnations. Plant Growth Regulation 18, 79–86

[CIT0048] ten Have A Woltering EJ 1997 Ethylene biosynthetic genes are differentially expressed during carnation (*Dianthus caryophyllus* L) flower senescence. Plant Molecular Biology 34, 89–97 9177315 10.1023/a:1005894703444

[CIT0049] Thomas CJR Smith AR Hall MA 1985 Partial purification of an ethylene-binding site from *Phaseolus vulgaris* L. cotyledons. Planta 164, 272–277 10.1007/BF00396092 24249571

[CIT0050] Thomas H Ougham HJ Wagstaff C Stead AD 2003 Defining senescence and death. Journal of Experimental Botany 54, 1127–1132 12654863 10.1093/jxb/erg133

[CIT0051] Thompson JE 1988 The molecular basis for membrane deterioration during senescence. In: Noodén LD Leopold AC, eds. Senescence and aging in plants . New York: Academic Press, 51–83

[CIT0052] Tieman DV Taylor MG Ciardi JA Klee HJ 2000 The tomato ethylene receptors NR and LeETR4 are negative regulators of ethylene response and exhibit functional compensation within a multigene family. Proceedings of the National Academy of Sciences, USA 97, 5663–5668 10.1073/pnas.090550597 PMC25885 10792050

[CIT0053] Tsuchisaka A Yu G Jin H Alonso JM Ecker JR Zhang X Gao S Theologis A 2009 A combinatorial interplay among the 1-aminocyclopropane-1-carboxylate isoforms regulates ethylene biosynthesis in Arabidopsis thaliana. Genetics 183, 979–1003 19752216 10.1534/genetics.109.107102 PMC2778992

[CIT0054] van Doorn WG van Meeteren U 2003 Flower opening and closure: a review. Journal of Experimental Botany 54, 1801–1812 12869518 10.1093/jxb/erg213

[CIT0055] van Zhong G Burns JK 2003 Profiling ethylene-regulated gene expression in *Arabidopsis thaliana* by microarray analysis. Plant Molecular Biology 53, 117–131 14756311 10.1023/b:plan.0000009270.81977.ef

[CIT0056] Verlinden S Boatright J Woodson WR 2002 Changes in ethylene responsiveness of senescence-related genes during carnation flower development. Physiologia Plantarum 116, 503–511

[CIT0057] Waki K Shibuya K Yoshioka T Hashiba T Satoh S 2001 Cloning of a cDNA encoding EIN3-like protein (DC-EIL1) and decrease in its mRNA level during senescence in carnation flower tissues. Journal of Experimental Botany 52, 377–379 11283184

[CIT0058] Wang H Woodson WR 1989 Reversible inhibition of ethylene action and interruption of petal senescence in carnation flowers by norbornadiene. Plant Physiology 89, 434–438 16666561 10.1104/pp.89.2.434 PMC1055859

[CIT0059] Woltering EJ Harkema H 1987 Amino-oxyacetic acid: analysis and toxicology. Acta Horticulturae 216, 273–280

[CIT0060] Woodson WR Lawton KA 1988 Ethylene-induced gene-expression in carnation petals—relationship to autocatalytic ethylene production and senescence. Plant Physiology 87, 498–503 16666171 10.1104/pp.87.2.498 PMC1054781

[CIT0061] Woodson WR Park KY Drory A Larsen PB Wang H 1992 Expression of ethylene biosynthetic pathway transcripts in senescing carnation flowers. Plant Physiology 99, 526–532 16668918 10.1104/pp.99.2.526 PMC1080495

[CIT0062] Yanagisawa S Yoo SD Sheen J 2003 Differential regulation of EIN3 stability by glucose and ethylene signalling in plants. Nature 425, 521–525 14523448 10.1038/nature01984

[CIT0063] Yang SF Hoffman NE 1984 Ethylene biosynthesis and its regulation in higher plants. Annual Review of Plant Physiology and Plant Molecular Biology 35, 155–189

