# Supplementary material for: Analysis of gene expression during the transition to climacteric phase in carnation flowers (Dianthus caryophyllus L.)
Source: J Exp Bot. 2013 Sep 28;64(16):4923–37. doi: 10.1093/jxb/ert281 (PMC3830478; doi:10.1093/jxb/ert281)
Supplement: Supplementary Data [file supp_64_16_4923__index.html]

Analysis of gene expression during the transition to climacteric phase in carnation flowers (Dianthus caryophyllus L.) — Analysis of gene expression during the transition to climacteric phase in carnation flowers (Dianthus caryophyllus L.) — Supplementary Data 

# Analysis of gene expression during the transition to climacteric phase in carnation flowers (*Dianthus caryophyllus* L.)

## Supplementary Data

Data files

**Files in this Data Supplement:**

- Supplementary Data - Supplementary Data
