# Supplementary material for: Analysis of gene expression during the transition to climacteric phase in carnation flowers (Dianthus caryophyllus L.)
Source: J Exp Bot. 2013 Sep 28;64(16):4923–37. doi: 10.1093/jxb/ert281 (PMC3830478; doi:10.1093/jxb/ert281)
Supplement: Supplementary Data [file supp_ert281_jexbot101691_file001.pdf]

Supplementary Fig. 1

Analysis of gene expression during the transition to climacteric phase in carnation flowers (*Dianthus caryophyllus* L.)

Byung-Chun In, Brad M. Binder, Tanya G. Falbel and Sara E. Patterson*

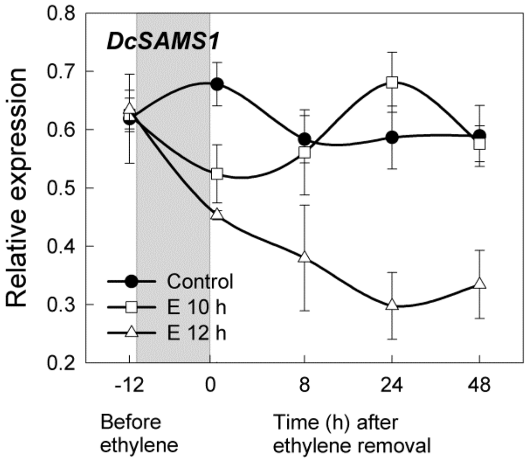

Supplementary Fig. 1. Expression of *DcSAMS1* in petals. Petals were treated with air only (control), 10 μL L^{-1} ethylene for 10 h (E 10 h) or 12 h (E 12 h). The grey boxes indicate the time period when the petals were exposed to ethylene. Petal samples were collected before ethylene treatment (-12 h) and 20 min, 8 h, 24 h, and 48 h after ethylene removal. Data represents the mean ±SE of five replicates.
